# Deficiency of emerin contributes differently to the pathogenesis of skeletal and cardiac muscles in *Lmna^H222P/H222P^* mutant mice

**DOI:** 10.1371/journal.pone.0221512

**Published:** 2019-08-20

**Authors:** Eiji Wada, Megumi Kato, Kaori Yamashita, Hiroko Kokuba, Wen-Chen Liang, Gisèle Bonne, Yukiko K. Hayashi

**Affiliations:** 1 Department of Pathophysiology, Tokyo Medical University, Tokyo, Japan; 2 Institute of Medical Science, Tokyo Medical University, Tokyo, Japan; 3 Department of Pediatrics, Kaohsiung Medical University Hospital, Kaohsiung Medical University, Kaohsiung, Taiwan; 4 Department of Pediatrics, School of Medicine, College of Medicine, Kaohsiung Medical University, Kaohsiung, Taiwan; 5 Sorbonne Université, Inserm UMRS 974, Center of Research in Myology, Paris, France; University of Minnesota Medical School, UNITED STATES

## Abstract

Laminopathies are tissue-selective diseases that affect differently in organ systems. Mutations in nuclear envelopes, emerin (*Emd*) and lamin A/C (*Lmna*) genes, cause clinically indistinguishable myopathy called Emery-Dreifuss muscular dystrophy (EDMD) and limb-girdle muscular dystrophy. Several murine models for EDMD have been generated; however, emerin-null (Emd) mice do not show obvious skeletal and cardiac muscle phenotypes, and *Lmna* ^H222P/H222P^ mutant (H222P) mice show only a mild phenotype in skeletal muscle when they already have severe cardiomyopathy. Thus, the underlying molecular mechanism of muscle involvement due to nuclear abnormalities is still unclarified. We generated double mutant (*Emd*^-/-^/*Lmna*^H222P/H222P^; EH) mice to characterize dystrophic changes and to elucidate interactions between emerin and lamin A/C in skeletal and cardiac muscles. As H222P mice, EH mice grow normally and have breeding productivity. EH mice showed severer muscle involvement compared with that of H222P mice which was an independent of cardiac abnormality at 12 weeks of age. Nuclear abnormalities, reduced muscle fiber size and increased fibrosis were prominent in EH mice. Roles of emerin and lamin A/C in satellite cells function and regeneration of muscle fiber were also evaluated by cardiotoxin-induced muscle injury. Delayed increases in *myog* and *myh3* expression were seen in both H222P and EH mice; however, the expression levels of those genes were similar with control and regenerated muscle fiber size was not different at day 7 after injury. These results indicate that EH mouse is a suitable model for studying skeletal muscle involvement, independent of cardiac function, in laminopathies and an interaction between emerin and lamin A/C in different tissues.

## Introduction

The nuclear envelope (NE) comprises the inner and outer nuclear membranes (INM and ONM, respectively) with disassembly of nuclear pore complexes and the underlying nuclear lamina network (type V intermediate filament proteins termed A-type and B-type lamins) in the inner membrane. The nuclear envelope has about 200 unique membrane proteins [1–3], which contribute to encapsulation of the nuclear genome, regulation of the cell cycle, and cytoskeletal organization; however, the functions of most NE proteins are still unknown [4].

The nuclear envelopathies are a group of disorders caused by mutations in genes encoding various nuclear envelope proteins. Emerin, which is a member of the LEM domain family, is highly conserved and ubiquitously expressed in all differentiated cells [5]. Mutations in *EMD* cause X-linked Emery-Dreifuss muscular dystrophy (EDMD) [6–8]. Mutations in *LMNA*, which encodes nuclear lamina proteins, the A-type lamins (lamins A and C, hereafter named lamin A/C), cause autosomal-dominant (AD) or less frequent recessive types of EDMD [9]. Clinical features of EDMD are characterized by progressive weakness in skeletal muscles, cardiomyopathy with conduction block, and early-onset joint contractures [10]. Mutations in *EMD* and *LMNA* also cause limb girdle muscular dystrophy [11, 12]. Moreover, mutations in *LNMA* are associated with a wide range of tissue-specific diseases called the laminopathies, including muscular dystrophy and cardiomyopathy, as well as peripheral neuropathy, familial partial lipodystrophy, and accelerated aging disorders, such as Hutchinson-Gilford progeria syndrome [13]. The underlying molecular mechanisms by which mutations in these genes encoding ubiquitously expressed NE proteins cause tissue-specific phenotypes have not been elucidated.

Several mouse models have been generated that demonstrate some aspects of the clinical phenotypes of nuclear envelopathy patients. Interestingly, a mouse with a knockout of the *Emd* gene (Emd mouse) is nearly normal and shows no overt dystrophic or cardiomyopathic phenotypes [14]. Only slight motor coordination problems, delayed muscle regeneration, and a mild atrioventricular conduction delay after 40 weeks of age have been reported [14, 15]. One possible reason for the absence of obvious phenotypes in Emd mice is the existence of a compensating factor. For example, recessive mutations in the *Tor1Aip1* gene, which encodes lamina-associated polypeptide 1 (LAP1), cause muscular dystrophy with cardiac involvement and dystonia [16, 17]. This INM protein interacts with emerin, and the conditional deletion of LAP1 from mouse skeletal muscle causes muscular dystrophy, whereas more severe phenotypes were observed coupled with emerin deficiency (emerin and muscle-specific LAP1 double-mutant mice) [18]. Two major mouse models of laminopathy, lamin A/C-null (*Lmna*^-/-^) mice [9] and H222P knock-in mice (*Lmna*^*H222P/H222P*^) carrying a missense mutation identified in a family with AD-EDMD [19, 20] have been generated to elucidate the pathomechanisms of laminopathy and EDMD. *Lmna*^-/-^ mice are not embryonic lethal but have a reduced growth rate, demonstrate dystrophic phenotypes in both skeletal and cardiac muscles, and die within 9 weeks of age [9, 21]. H222P mice demonstrate mild muscular dystrophy, left ventricular dilatation, and conduction defects in adulthood, resulting in death by 9 months of age in males. These mice represent a good model for human AD-EDMD [20]. The mechanisms involved in cardiomyopathies in H222P mice have been studied [22–29], and potential targets for drug therapy have been reported [30–33]. Despite the severe cardiac phenotypes in adult H222P mice, the histology and function of cardiac abnormalities are mild at early ages, and significant dystrophic pathology in skeletal muscle appears at a late stage (at 6 months of age) of the disorder [20, 34].

Nuclear membrane proteins are suggested to structurally associate with each other to maintain their normal cellular functions. The direct interaction between emerin and lamin A and C (lamin A/C) have been reported [35, 36], and deficiency or dysfunction of these proteins may result in abnormalities in nuclear structure, and alterations in gene expression and signal transduction pathways [37, 38]. Multisystem involvement in a family harboring both *EMD* and *LMNA* mutations was previously reported, which highlighted the crucial role of the interaction between emerin and lamin A/C [39]. We hypothesized that emerin deficiency affects on skeletal and cardiac muscles in H222P mice. In this study, we produced *Emd*^-/-^/*Lmna*^*H222P/H222P*^ double-mutant (EH) mice to elucidate the interactive functions of emerin and lamin A/C, and compared their pathological changes, particularly of the skeletal muscle, with those of mouse models of EDMD.

## Materials and methods

### Mice

Emd and H222P mice were generated as previously described [14, 20]. As Emd mice were on a C57BL/6J background, H222P mice were backcrossed on the same strain, and then EH (Emd/222P) mice were produced. Genotyping was performed by PCR using specific primer sets as described previously [14, 20]. All mice were maintained in a specific pathogen-free facility with 12-h/12-h light/dark cycles. Male mice were weighed every week and used for further analysis. Institutional Animal Care & Use Committee in Tokyo Medical University animal facility approved all experiments performed in this study (number H30-0036, H31-0075).

### Transthoracic echocardiography

Mice (n = 8–9 in each group) were anesthetized with 3% isoflurane until their heart rate stabilized at 400 to 500 beats per minute, and then they were sedated with 1% isoflurane continuously. Long axis M-mode images were recorded at the papillary muscle level using a 15.3 MHz transducer with ARIETTA prologue (Hitachi, Ltd.). The left ventricular ejection fraction (LVEF) was calculated as follows: LVEF (%) = [(LVEDV–LVESV)/LVEDV] ×100, in which LVEDV is left ventricular end-diastolic volume, and LVESV is left ventricular end-systolic volume.

### Wheel running and exhaustion treadmill

Muscle functions were evaluated using a voluntary running wheel and a treadmill. Mice (12 weeks of age, n = 7 in each group) were acclimatized to the running wheel cage with a digital counter for 3 days, and data of daily wheel rotations were collected for the following 4 days. After testing voluntary running activity, mice were housed in a normal cage for 2 days. The same mice were used for exhaustion treadmill analysis, which was carried out using a six-lane motorized treadmill supplied with shocker plates. The protocol was modified as previously reported [40]. Briefly, the test was started at an inclination of 0° at 5 m/min for 5 min. The speed was gradually increased by 1 m/min every minute until the mouse remained behind the shocker plate without attempting to return to running on the treadmill within 20 seconds. Three tests were performed on the same mouse, with 2 days in between each test. The first test was used as acclimatization, and the average of the following 2 tests was used as the result of each mouse.

### Serum biochemical analysis

Serum samples were isolated from the blood by incubation for 2 h at 4°C, followed by centrifugation at 8,000 rpm for 15 min. Serum creatine kinase (CK) levels were measured using a biochemistry automatic analyzer (model 7180; Hitachi High-Tech).

### Histopathological analysis

The heart and skeletal muscles (quadriceps, gastrocnemius, soleus, paravertebral, abdomen, and diaphragm) were isolated from mice at 12 and 30 weeks of age and were frozen in isopentane cooled in liquid nitrogen. Transverse 10-μm-thick cryosections were collected and stained with hematoxylin and eosin (H&E). For immunohistochemistry, cryosections were fixed with 4% paraformaldehyde/PBS. After blocking with 2% BSA/PBS, samples were incubated with primary antibodies against periostin (no. NBP1-30042, Novus Biologicals), laminin α2 (no. ALX-804-190, Enzo Life Sciences), lamin A/C (no. 4C11, Cell Signaling), LAP2α (no. ab5162, Abcam), nesprin 1 (no. ab192234, Abcam) and embryonic myosin heavy chain (eMyHC, no. F1.652, Developmental Studies Hybridoma Bank) at 37°C for 1 h. Alexa Fluor 488 or 568 secondary antibodies (1:1,000; Thermo Fisher Scientific) with DAPI solution was used for detection. Muscle membrane and nuclei were stained with laminin α2 and DAPI and were captured using an IN Cell Analyzer 2200 imaging system. Fiber diameters (the minor axis or area) and the percentage of fibers with internal nuclei were automatically calculated by the IN Cell Developer Toolbox software (GE Healthcare). The percentage area of regenerating fibers in the soleus muscle was detected by the eMyHC antibody and calculated using NIH ImageJ software. To detect degenerating muscle fibers with permeable membranes, mice were intraperitoneally injected with 1% Evans Blue Dye (EBD, w/v) in saline [41]. Cryosections were obtained and images were acquired using a fluorescence microscope.

### RNA isolation and quantitative real-time RT-PCR analysis

Total RNA was extracted from the tibialis anterior (TA), soleus, and cardiac muscles using the PureLink RNA mini kit (Life Technologies), and cDNA was synthesized using superscript VILO cDNA synthesis kit (Invitrogen) according to the manufacturer’s instructions. Real-time quantitative RT-PCR was performed on the QuantStudio 3 Real-Time PCR system using SYBR green master mix (Applied Biosystems). The primer sequences used for gene expression analyses are listed in S1 Table. All results were normalized using the endogenous gene *Gapdh*, and fold changes in gene expression were determined by the ΔΔCt method. Data were expressed as the fold-increase versus the values of wild type (WT) mice.

### Protein extraction and western blot analysis

Muscle samples (quadriceps, soleus, and heart) were homogenized in RIPA buffer containing protease inhibitors and phosphatase inhibitors (Roche). After centrifugation at 15,000 rpm for 20 min, supernatants were collected and mixed with sample buffer solution (WAKO). Extracted proteins (60 μg) were loaded onto 5%–20% or 10%–20% gradient SDS-PAGE gels (WAKO) and transferred to PVDF membranes using a Trans-Blot Turbo system (Bio-Rad). The membranes were blotted with primary antibodies against periostin (no. NBP1-30042, Novus Biologicals), emerin (no. ab156871, Abcam), lamin A/C (no. 4C11, Cell Signaling), LAP2α (no. ab5162, Abcam), lamin B1 (no. 66095-1-lg, Proteintech), or GAPDH (no. ab8245, Abcam). Secondary antibodies were horseradish peroxidase-conjugated secondary antibodies (Thermo Fisher Scientific), and ECL substrate solution was used for visualization by a ChemiDoc imager (Bio-Rad). The band intensities of the periostin protein were normalized by the band intensity of GAPDH.

### Electron microscopy

Soleus muscle from H222P and EH mice were fixed with 2.5% glutaraldehyde in 0.1 M sodium cacodylate buffer for 2 h. After washing in cacodylate buffer, the specimens were postfixed in 1% osmium tetroxide for 1 h, dehydrated in graded series of ethanol, and embedded in Epon (Nisshin EM Co., Ltd.). The ultrathin sections were stained with lead nitrate and uranyl acetate and observed using a JEOL JEM-1200EX II electron microscope (JEOL Ltd.).

### Cardiotoxin injection

A single injection of cardiotoxin (CTX, 10 μmol/L in 100 μL saline) was administered into the TA muscles of mice at 12 weeks of age. Muscle samples were obtained at 3, 5, and 7 days after the injection (n = 4 in each group).

### Statistical analysis

Results were expressed as means±SD. SPSS Statistics software was used to perform statistical analysis, and differences were determined by one-way ANOVA with the post-hoc Tukey multiple comparison test. Statistical tests were two-sided, and *P*-values ≤ 0.05 were considered to indicate a statistically significant difference between two groups.

## Results

### Phenotype analysis of EH mice

**Fig 1:**
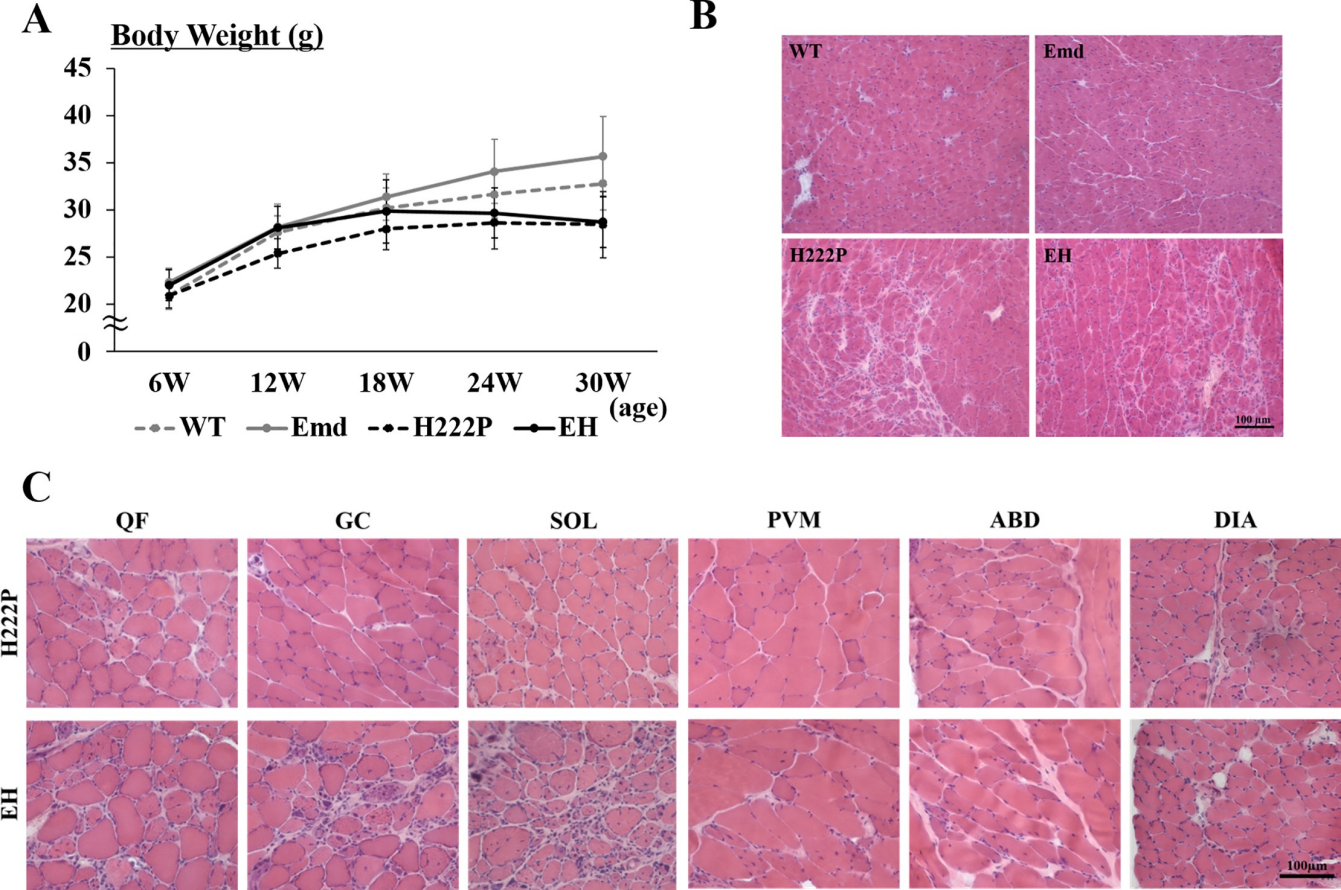
Growth curves and cardiac and skeletal muscle histology of mice at 30 weeks of age. (A) Body weights of male mice at 6, 12, 18, 24, and 30 weeks of age. WT: grey dotted line (n = 17); Emd: grey line (n = 17), H222P: black dotted line (n = 13) and EH: black line (n = 17) mice. (B) Cardiac histology of mice at 30 weeks of age. (C) Skeletal muscle histology of H222P and EH mice at 30 weeks of age. quadriceps: QF; gastrocnemius: GC; soleus: SOL; paravertebral: PVM; abdomen: ABD; and diaphragm: DIA.

To assess the effects of emerin deficiency in H222P mice, we measured body weight and observed phenotypes of EH mice in a standard cage. The original H222P mice were backcrossed to the C57BL/6J strain. Emd, H222P, and EH mice were indistinguishable from WT mice and did not show any abnormal behavior in a standard cage during their childhood. Whereas H222P mice had relatively lower body weights than WT mice, EH mice had body weights comparable to WT mice before 18 weeks of age. EH mice did not have morphological abnormalities in different organ systems, such as the kidneys, lungs and reproductive organs; however, they showed gradual body weight loss after 18 weeks of age (Fig 1A). Similarly to H222P mice, EH mice demonstrated an abnormal appearance, such as scoliosis and rapid breathing, and died around 6 months of age mainly due to cardiac failure. As previously reported, H222P mice demonstrate severe pathological changes in the cardiac muscle at 30 weeks of age, and cardiac muscle from EH mice show similar histological changes from that of H222P mice at the same age (Fig 1B). At 30 weeks of age, muscle pathology in different parts of skeletal muscle regions was exacerbated in EH mice compared with that of H222P mice (Fig 1C).

### No difference in cardiac function and morphology between H222P and EH mice at 12 weeks of age

**Fig 2:**
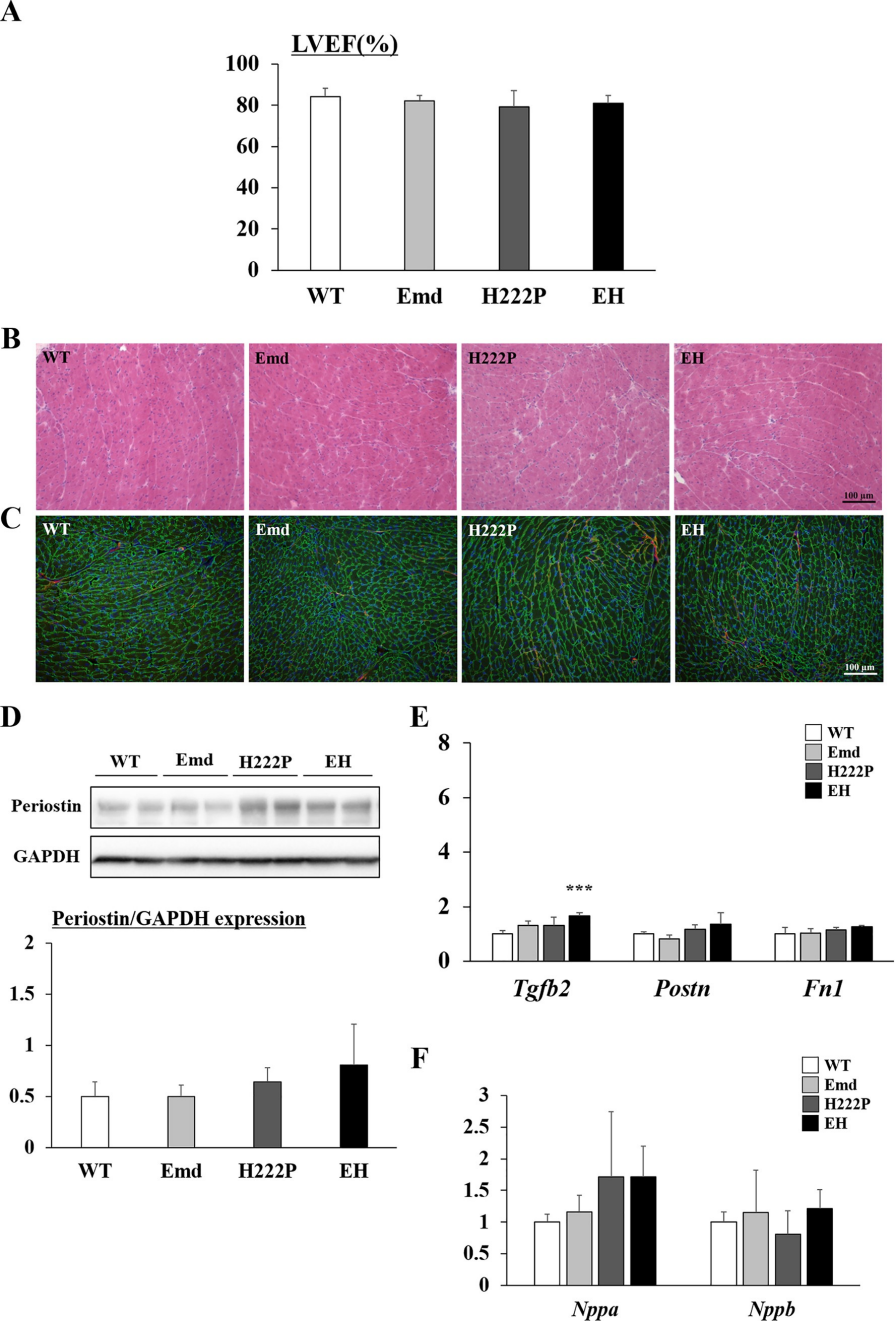
Cardiac muscle phenotypes of mice at 12 weeks of age. (A) Evaluation of cardiac function by transthoracic echocardiographic analyses. Left ventricular ejection fractions (LVEFs) are shown (n = 8–9). (B) H&E staining of cryosections from cardiac muscle. (C) Immunostaining of laminin ɑ2 (green) and periostin (red) with DAPI (blue) from cardiac muscle. (D) Western blot analyses of periostin. The graph shows the quantification of periostin levels normalized to GAPDH (n = 3). (E) qPCR analyses of *Tgfb2* (TGF β2), *Postn* (periostin), and *Fn1* (fibronectin) mRNA levels in cardiac muscle (n = 4). (F) qPCR analyses of *Nppa* (natriuretic peptide A) and *Nppb* (natriuretic peptide B) mRNA levels in cardiac muscle (n = 4). In qPCR analyses, data were normalized by *Gapdh* mRNA and expressed as fold increases from WT mice. ***P<0.001 compared with WT mice.

We next assessed the cardiac function in H222P and EH mice. At 12 weeks of age, there was no difference in LVEF among genotypes (Fig 2A). To confirm that cardiac muscle from EH mice was maintained at 12 weeks of age, we analyzed histological changes in cardiac muscle. At 12 weeks of age, there were no histological abnormalities in any of the genotypes, and fibrosis was not increased both in H222P and EH cardiac muscle (Fig 2B and 2C). In addition, levels of the fibrotic marker periostin were similar in H222P and EH mice by western blotting (Fig 2D). The gene expression level of *Tgfb2* in EH mice was slightly increased compared with that of WT mice, but not with that of H222P mice (Fig 2E), whereas the gene expression of periostin and fibronectin were similar in all genotypes. No statistical differences in gene expression levels of *Nppa* and *Nppb* also supported that cardiac function was still maintained in H222P and EH mice at this age (Fig 2F).

### Reduced skeletal muscle function of EH mice at 12 weeks of age

**Fig 3:**
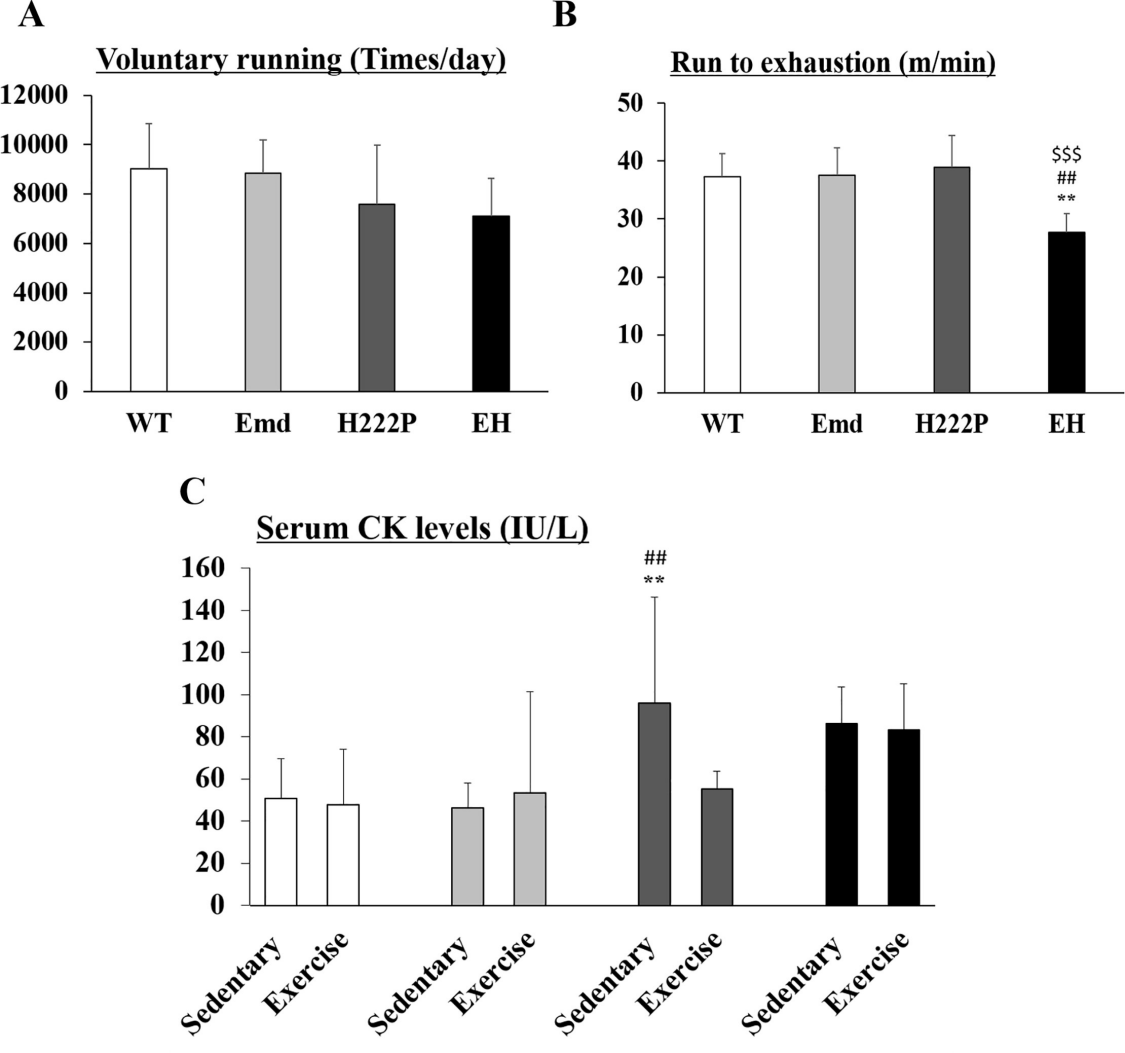
Skeletal muscle functions of mice at 12 weeks of age. (A) Average daily activity levels analyzed using a voluntary running wheel. Data were expressed as revolutions per day (n = 7). (B) Treadmill running to exhaustion. The average maximum speeds at which mice could not continue running are shown (n = 7). (C) Serum CK levels from sedentary and exercised mice (n = 7–8). **P<0.01 compared with WT mice; ##*P*<0.01 compared with Emd mice; $$$*P*<0.001 compared with H222P mice.

We next assessed the skeletal function in H222P and EH mice at 12 weeks of age. To test their voluntary running performance, the wheel-running test was performed. After acclimation to the wheel-running device, the average of 4 days of testing was calculated. WT and Emd mice ran on the wheel about 9,000 times per day (9,007±1,854 and 8,845±1,342 times per day, respectively), and H222P and EH mice ran for slightly less (7,585±2,385 and 7,107±1,530 times per day, respectively) than WT mice but the difference was not statistically significant (Fig 3A). Mice were subjected to the treadmill exhaustion test after finishing the wheel running test. They were placed and familiarized in the treadmill before the start of data collection. The results from WT, Emd, and H222P mice were not different (37.4±3.9, 37.5±4.8, and 38.9±5.5 m/min, respectively); however, EH mice had significantly less capacity to run fast (27.6±3.3 m/min) (Fig 3B). In detail, EH mice tried to escape from the shocker plate and go back onto the treadmill several times, but they failed to do so. One day after the exercise tests, mice were sacrificed and serum CK levels were measured. Compared with the CK levels from sedentary mice of each genotype, there were no significant increases in CK levels after the exercise tests in all genotypes (Fig 3C).

### Severer skeletal muscle phenotypes in EH mice at 12 weeks of age

**Fig 4:**
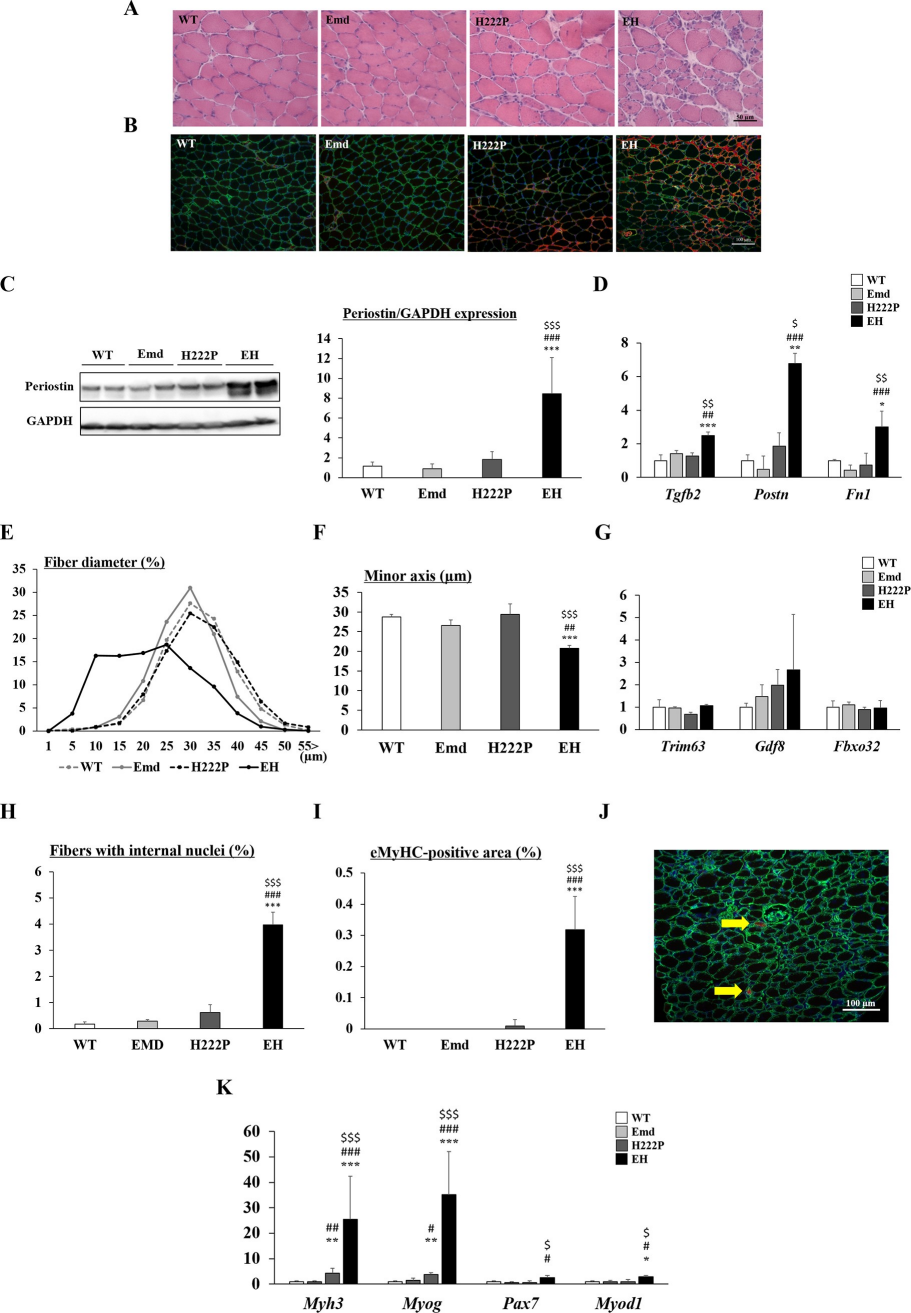
Skeletal muscle pathology of mice at 12 weeks of age. (A) H&E staining of cryosections and (B) immunostaining of laminin ɑ2 (green) and periostin (red) with DAPI (blue) from soleus muscle. (C) Western blot analyses of periostin from soleus muscle. The graph shows quantification of periostin levels normalized to GAPDH (n = 4). (D) qPCR analyses of *Tgfb2* (TGF β2), *Postn* (periostin), and *Fn1* (fibronectin) mRNA levels in soleus muscle (n = 4). (E) Histogram of muscle fiber diameters in soleus muscle. Data are expressed as a percentage of total fibers. (F) The average muscle fiber size by measuring the minor axis of fibers (n = 3–4). (G) qPCR analyses of muscle atrophy-associated genes, *Trim63* (MuRF-1), *Gdf8* (myostatin) and *Fbxo32* (atrogin-1) (n = 4). (H) The percentage of fibers with internal nuclei per total fibers (n = 4). (I) The percentage of embryonic myosin heavy chain (eMyHC)-positive regenerating fiber area per total muscle area (n = 4). (J) Immunostaining of laminin ɑ2 (green) and EBD (red) with DAPI (blue) from soleus muscle. Yellow arrows show the presence of EBD-positive necrotic myofibers. (K) qPCR analyses of muscle regeneration-related genes, *Myh3* (eMyHC), *Myog* (myogenin), *Pax7* (Pax7) and *Myod1* (MyoD) (n = 4). In qPCR analyses, data were normalized by *Gapdh* mRNA and expressed as fold increases from WT mice. *P<0.05, **P<0.01, and ***P<0.001 compared with WT mice; #*P*<0.05, ##*P*<0.01, and ###*P*<0.001 compared with Emd mice; $*P*<0.05, $$*P*<0.01, and $$$*P*<0.001 compared with H222P mice.

To analyze the contribution of emerin deficiency in H222P mice, particularly of the reduced skeletal muscle function, we evaluated different parts of the skeletal muscle by H&E staining at 12 weeks of age. Moreover, dystrophic changes in skeletal muscle were observed even at the younger age of 12 weeks in EH mice (Fig 4A). Soleus muscle was used for further evaluation of muscle pathology. The protein levels of periostin were significantly increased in EH mice and was slightly, but not significantly increased in H222P mice (Fig 4B and 4C). This result was consistent with the upregulation of the expression of fibrosis-associated genes, such as *Tgfb2*, *Postn*, and *Fn1* (Fig 4D). Muscle fiber size, which is determined by the minor axis of muscle fibers, was significantly reduced in EH mice whereas Emd and H222P mice maintained the size at 12 weeks of age (Fig 4E and 4F). Muscle atrophy-associated gene expression levels were determined by qPCR; however, there were no significant differences in the levels of *Gdf8* (myostatin), *Trim63* (MuRF-1), or *Fbxo32* (atrogin-1) in EH mice (Fig 4G). Significant increases in the percentage of muscle fibers with internal nuclei (Fig 4H) and eMyHC-positive regenerating fibers representing muscle damage in EH mice (Fig 4I) were observed, whereas EBD-positive necrotic fibers were rarely seen (Fig 4J). In EH muscle, gene expression levels of *Pax7*, *Myod1*, *Myog*, and *Myh3* were statistically higher, and H222P mice also had increased *Myog* and *Myh3* expression levels in skeletal muscle compared with WT and Emd mice (Fig 4K).

### Abnormal nuclei in cardiac and skeletal muscles of H222P and EH mice

**Fig 5:**
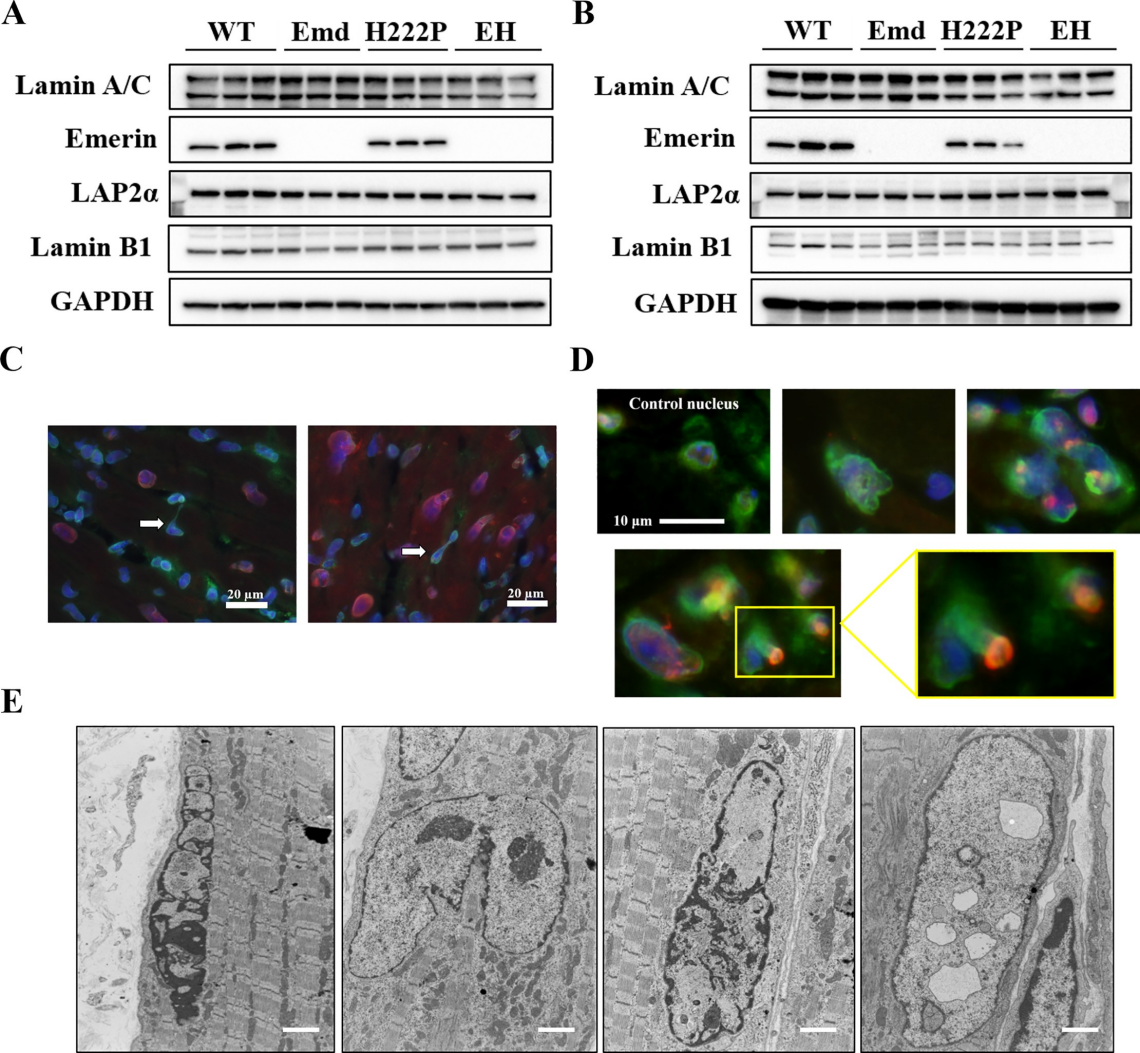
Nuclear changes in cardiac and skeletal muscles. (A and B) Western blot analyses of lamin A/C, emerin, LAP2α, and lamin B1 in (A) cardiac muscle and (B) skeletal muscle from WT, Emd, H222P, and EH mice. GAPDH was used as an internal control to ensure that equal sample volumes were applied. (C) Immunodetection of cardiac nuclei with abnormal shapes in EH mice. Lamin A/C (green) and either nesprin 1 (right, red) or LAP2α (left, red) were colocalized with DAPI-positive nuclei (blue). White arrows indicated elongated nuclei. (D) Immunodetection of nuclei with abnormal shapes in the soleus muscle from EH mice at 12 weeks of age. Bottom panels show that either nesprin 1 (upper, red) or LAP2α (lower, red) was mislocalized in lamin A/C (green) and DAPI-positive nuclei (blue). This mislocalization was also seen in lamin A/C and nesprin 1 double-stained cryosections in skeletal muscle from EH mice. (E) Electron microscopic observation of abnormal myonuclei from EH mice at 8 weeks of age. The irregular localization of highly condensed heterochromatin (left), enlarged and dysmorphic nuclei (middle left and middle right), and abnormal intranuclear vacuoles (right); scale bar = 2 μm.

To elucidate the interactive functions of emerin and lamin A/C in EH mice, the protein levels of nuclear membranes (lamin A/C, emerin, lamin B1, and LAP2α) were analyzed by western blotting. In total cardiac and skeletal (quadriceps) muscle samples, the expression of membrane proteins was not notably different among the genotypes at the age of 12 weeks, whereas emerin was similarly expressed only in WT and H222P mice (Fig 5A and 5B). Therefore, emerin deficits and mutations in lamin A/C in EH mice did not affect the protein expression levels of lamin A/C, lamin B1, and LAP2α. However, analysis of cardiac and skeletal muscles by immunohistochemistry demonstrated that nuclei with abnormal shapes were detected in both H222P and EH mice at 12 weeks of age (Fig 5C and 5D). In skeletal muscle, abnormal nuclei were frequently seen around dystrophic or atrophied fibers. The size of the abnormal nuclei was variable, and some nuclei were elongated or connected to each other. On transverse cryosection microscopy, the mislocalization of other nuclear membrane proteins (LAP2α and nesprin 1) was detected in the abnormal nuclei in both H222P and EH skeletal muscle (Fig 5D). The ultrastructure of nuclei in soleus muscle from H222P and EH mice at 8 weeks of age was observed in the longitudinal direction using electron microscopy. Morphological changes in nuclei were already seen in the sample from 8 weeks of age, even though both INM and ONM were distinct. Remarkable pathological phenotypes were as follows: irregular localization of highly condensed heterochromatin, tandem myonuclei, enlargement, and disruption of nuclei, which were observed in both H222P and EH mice (Fig 5E). In addition, intranuclear vacuoles were observed only in EH skeletal muscle (Fig 5E, right).

### Persistence of regenerative capacity after injury

**Fig 6:**
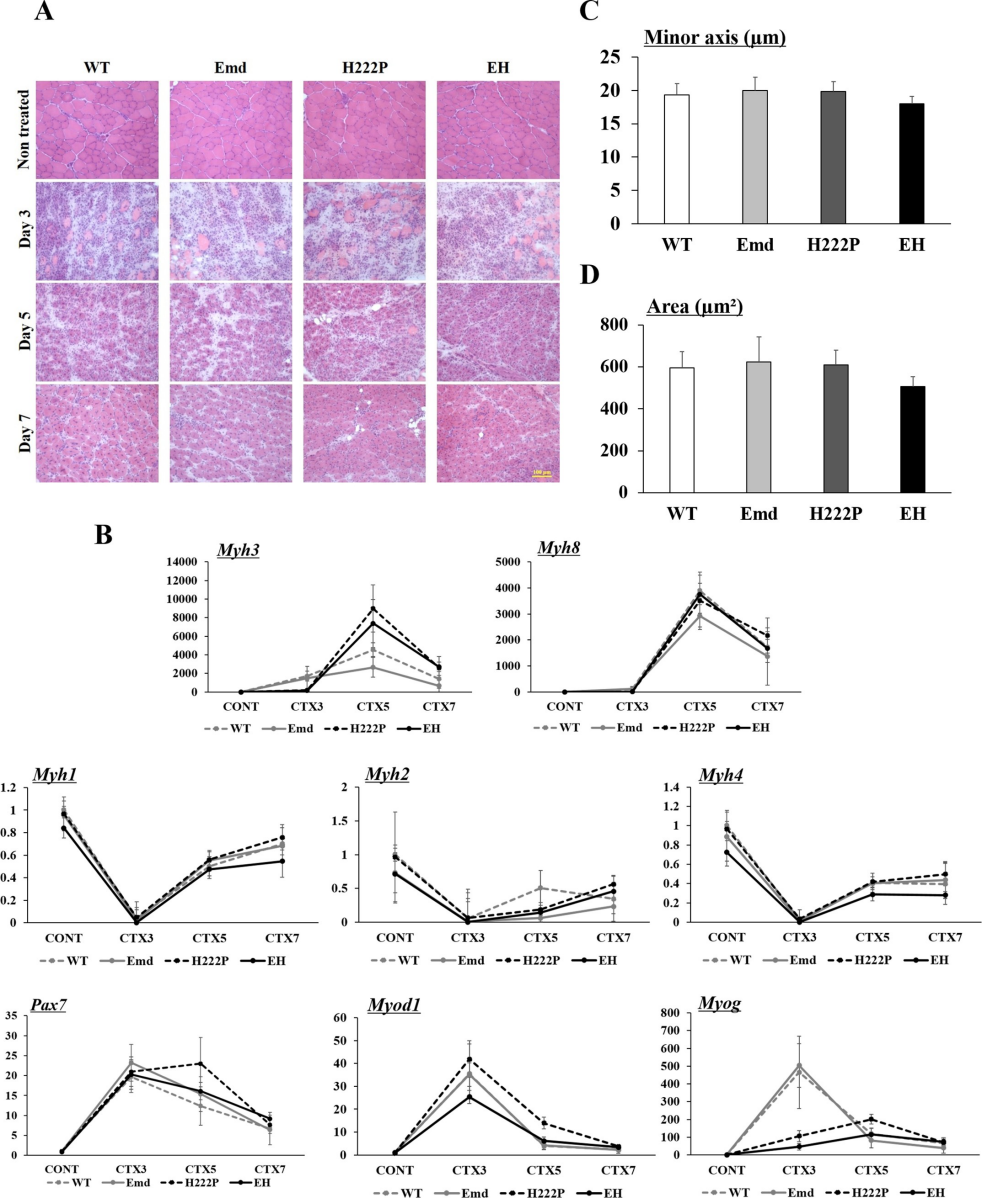
Muscle regeneration from CTX-induced muscle injury. (A) H&E staining of cryosections from the TA muscle of nontreated, and 3 days, 5 days, and 7 days after CTX injection (n = 4). (B) qPCR analyses of *Myh3*, *Myh8*, *Myh1*, *Myh2*, *Myh4*, *Myog*, *Pax7*, and *Myod1* mRNA levels from the muscle of nontreated (CONT), and 3 days, 5 days, and 7 days after CTX injection. Data were normalized by *Gapdh* mRNA and expressed as fold increase of WT (CONT) mice. (C and D) The average regenerated muscle fiber size analyzed by measuring (C) the minor axis and (D) the area from the TA muscle of mice 7 days after CTX injection.

To analyze satellite cell function *in vivo*, CTX was injected into TA muscle, and the process of muscle regeneration was observed at 3, 5, and 7 days postinjury (Fig 6A). The H&E stained TA muscle contained myofiber necrosis and inflammatory cell infiltration on day 3 after CTX injection in all mice. There were no histological differences among the genotypes; however, the expression of *Myog* and *Myh3* was delayed in H222P and EH mice (Fig 6B). The expression of these genes was upregulated in these genotypes on day 5 postinjury when many small regenerating fibers were present. Most of the damaged areas were replaced with newly formed regenerated muscle fibers with internal nuclei by 7 days after CTX injection. On day 7, the expression levels of genes associated with satellite cell function and muscle regeneration were similar in all mice (Fig 6B). To analyze muscle growth following postinjury, 500 to 600 regenerating fibers from each TA muscle were measured. Both the minor axis and area of regenerating fibers were not significantly different among the genotypes (Fig 6C and 6D). Throughout the regeneration, the gene expression levels of *Myh1* (Type 2X/D), *Myh2* (Type 2A), and *Myh4* (Type 2B), which encode myosin heavy chains in TA muscle, were also similar (Fig 6B).

## Discussion

Mutations in genes encoding NE proteins result in distinct disease phenotypes; however, interactions of NE proteins are thought to be an important factor for understanding the tissue-specific phenotypes. We predicted that the deficiency of emerin in a mouse model carrying a *Lmna* mutation may cause pronounced abnormalities in both skeletal and cardiac muscles. To test our hypothesis, we generated *Emd*^-/-^/*Lmna*^*H222P/H222P*^ double-mutant (EH) mice.

As described previously, Emd mice did not demonstrate any abnormalities in cardiac and skeletal muscles at 30 weeks of age [14], whereas dystrophic changes in cardiac and skeletal muscles were observed in H222P mice at the same age. H222P mice have a late onset and slowly progressive cardiac symptoms, and therefore they are a suitable model for studying pathogenic mechanisms in EDMD, particularly in the heart [20]. In our study, dystrophic phenotypes in skeletal muscle from H222P mice were detected at 30 weeks of age. A previous study also reports that H222P mice at 20 weeks of age have smaller fiber size and increased numbers of regenerated myofibers [42]. Although H222P mice present a later onset as well as slower disease progression in both the skeletal and cardiac muscles, their pathophysiological phenotypes at a younger age have not been fully analyzed. Our data showed that major pathological and physiological changes were not observed at 12 weeks of age; however, the significant upregulation of *Myh3* and *Myog* gene expression and an increase in serum CK levels in H222P mice demonstrated that the onset of skeletal muscle involvement in H222P mice is around this age.

EH mice are not embryonic lethal and have the ability to reproduce. No morphological abnormalities are seen in the brain, bone, and internal organs. Similarly to WT and Emd mice, EH mice gained body weight normally up to 18 weeks of age; however, it gradually reduced in conjunction with cardiac dysfunction. Histopathological observations demonstrated that EH mice had similar pathological changes in cardiac muscle with H222P mice at 30 weeks of age; prominent fibrotic areas were observed compared with WT and Emd mice. In contrast to cardiac muscle, deficits of emerin under mutations in lamin A/C prominently affect skeletal muscle at 30 weeks of age. Pathological changes in various regions of skeletal muscles in EH mice, particularly in the quadriceps, gastrocnemius, and soleus, were more exacerbated compared with those of H222P mice; however, the diaphragm had only minimum changes in H222P and EH mice. It is possible that different background strains may modify pathological changes in H222P mice [20, 34]. Skeletal muscle pathology in EH mice included a wide variation in fiber size, an increased number of small atrophic fibers, fibers with internal nuclei, and increased fibrotic area, which are similar to the dystrophic features presenting in human EDMD [11].

We clearly demonstrated that EH mice at 12 weeks of age did not show any major pathological or physiological changes in cardiac muscle. Periostin is highly accumulated in the fibrotic areas of the extracellular matrix of dystrophic muscles, and is a reliable marker to quantify dystrophic fibrosis by immunohistology and western blotting [43]. In cardiac muscle at 12 weeks of age, periostin levels in both H222P and EH mice were not different from those in WT and Emd mice by western blotting, which was consistent with the results of qPCR analysis. Although the upregulation of *Tgfb2* signaling was reported in H222P cardiac muscle at a late stage [44], it was upregulated in EH cardiac muscle compared with WT mice but not H222P mice at 12 weeks of age. Cardiac function was still maintained in EH mice at 12 weeks of age; therefore, the deficiency of emerin in cardiac muscle did not contribute to the exacerbation of cardiac dysfunction in H222P mice.

In contrast to cardiac muscle, skeletal muscle was exacerbated in EH mice at 12 weeks of age. The substantial increase in fibrosis and regenerating areas and decrease in fiber size in EH mice demonstrated that a single mutation of emerin or lamin A/C is insufficient to trigger skeletal muscle pathology in murine models of EDMD. As EH mice grow normally and have breeding ability, the voluntary exercise level measured using a running wheel was also normal at 12 weeks of age. To test muscle performance, the treadmill exhaustion procedure was used to analyze exercise capacity and endurance. EH mice could not keep up with the gradually increasing speed and could not run fast, whereas H222P mice had similar exercise capacity to that of WT and Emd mice. Cardiac pathophysiology was similar in H222P and EH mice, and hence the reduced exercise capacity of EH mice is thought to be mainly due to skeletal muscle abnormalities.

In EH mice at 12 weeks of age, the expression of NE proteins, such as lamin A/C, emerin, LAP2α, and lamin B1, were detected in similar amounts in skeletal and cardiac muscles compared with WT, Emd, and H222P mice. A typical cellular hallmark of EDMD is the presence of abnormal nuclear morphology. At 12 weeks of age, a few abnormal nuclei were observed in cardiac and skeletal muscles EH mice. Elongated nuclei were detected in cardiac muscle, which was also reported in cardiomyocytes from H222P and *Lmna*^*–/–*^mice [20, 45]. In skeletal muscle, morphological abnormalities were classified as enlarged, elongated, wrinkled, and deformed nuclei, which were also observed in both skeletal muscle from laminopathy patients and *LMNA*-mutant human induced pluripotent stem cell-derived inducible myogenic cells [46, 47]. In addition, the mislocalization of lamin A/C and NE proteins (LAP2α and nesprin 1) were detected in the nuclei of both H222P and EH skeletal muscle. The ultrastructure of nuclei from skeletal muscle analyzed by electron microscopy demonstrated that several pathological changes were observed in myonuclei. Highly condensed heterochromatin and morphological changes of nuclei were detected whereas the surrounding myofibrils were not disrupted. Interestingly, a few myonuclei from EH skeletal muscle but not from H222P skeletal muscle contained intranuclear vacuolar structures. We previously reported that unique perinuclear vacuolar structures associated with autophagy were detected in the skeletal and cardiac muscles from laminopathy patients and H222P mice [47, 48]. Similar intranuclear vacuoles were seen in skeletal muscles from AD-EDMD [47]. The presence of inner nuclear vacuolar structures in skeletal muscle from EH mice could be a result of the more fragile lamina structure, which occurs similarly in laminopathy and EDMD patients.

The crucial difference between skeletal and cardiac muscles is the presence of muscle stem cells called satellite cells. Morphological abnormalities of satellite cells from human EDMD and H222P mice were previously reported [20, 47]; however, the function of satellite cells was not fully analyzed. We evaluated satellite cell function by the *in vivo* muscle degeneration/regeneration assay using CTX injection into TA muscle. During regeneration, WT and Emd mice had similar gene expression patterns of *Myog*, *Pax7*, and *Myod1*. These gene expression patterns were prominently different in H222P and EH mice compared with WT mice on day 3 and day 5 after CTX injection. Delayed upregulation of the *Myh3* gene was also demonstrated in both H222P and EH mice; however, the expression patterns of genes associated with muscle fiber types (*Myh1*, *Myh2*, and *Myh4*) were similar among all genotypes during regeneration. On day 7, regenerating muscle fiber size was not different among all genotypes, and therefore satellite cell function and muscle maturation were maintained in murine models of EDMD at 12 weeks of age.

The pathogenic mechanisms of highly tissue-specific phenotypes involved in the nuclear envelopathies, particularly those with lamin A/C mutations, have remained unknown. Several nonexclusive hypotheses have been proposed. The ‘structural hypothesis’ suggests that the loss of structural functions of lamin A/C leads to a fragile nuclear membrane against mechanical stress [49, 50]. The ‘gene regulation hypothesis’ also proposes that tissue-specific transcription of transcription factor-regulated genes that interact with emerin or lamin A/C is affected and the localization and stability of these factors in the nuclei are altered [51, 52]. Another hypothesis is the ‘stem cell function hypothesis’, which proposes that the function of stem cells may be impaired by mutations in NE proteins [53]. Our results proposed that the interaction between NE proteins may be altered in different organ systems, and therefore, altered nuclear membrane structures owing to the presence of mutant NE proteins may cause impaired mechanotransduction and vulnerability that results in damage to nuclei.

In summary, EH mice show the progression of muscular dystrophy before cardiac dysfunction appears similar to EDMD patients, and therefore the molecular mechanisms of skeletal muscle involvement in EDMD, independent of cardiac function, can be studied in more detail. Our data clearly suggested that emerin and lamin A/C undergo crosstalk to accomplish distinct functions in cardiac and skeletal muscles. Emerin has been implicated to contribute to skeletal or cardiac muscle pathology in a manner different to that of lamin A/C. Tissue specific or non-specific proteins may participate in the interactive functions of emerin and lamin A/C in different tissues. Further studies are required to clarify the molecular mechanisms of abnormalities in skeletal and cardiac muscle in young and adult H222P and EH mice. EH mice, in addition to H222P mice, provide important features for understanding the tissue-specific pathogenesis of the nuclear envelopathies.

## Supporting information

S1 Table Primer sequences used for quantification of gene expression. (DOCX)
