# Supplementary figures and images for: Deficiency of emerin contributes differently to the pathogenesis of skeletal and cardiac muscles in LmnaH222P/H222P mutant mice
Source: PLoS One. 2019 Aug 20;14(8):e0221512. doi: 10.1371/journal.pone.0221512 (PMC6701770; doi:10.1371/journal.pone.0221512)

**Supporting table**


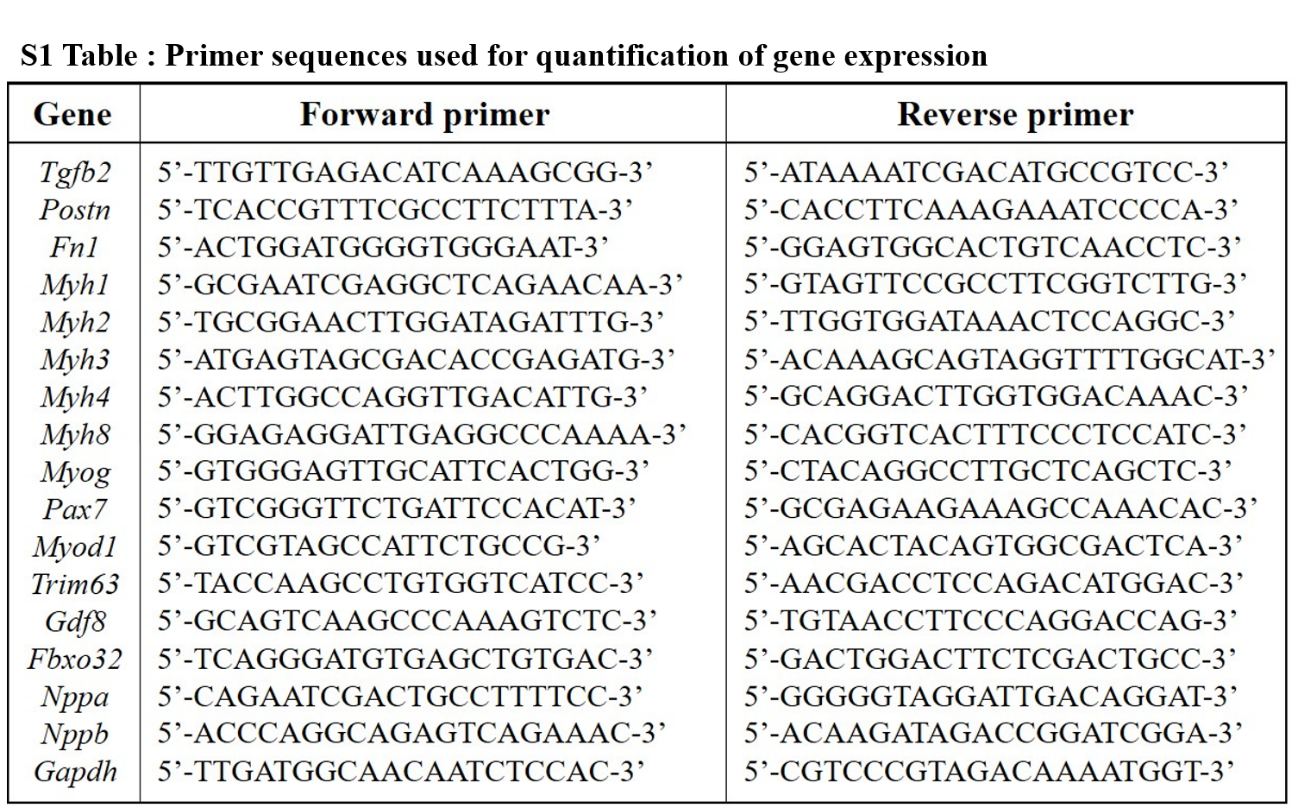

Supplement: S1 Table — (DOCX) [file pone.0221512.s001.docx]
